# The Therapeutic Challenge of Disseminated Bone Marrow Metastasis From HR-Positive HER2-Negative Breast Cancer: Case Report and Review of the Literature

**DOI:** 10.3389/fonc.2021.651723

**Published:** 2021-10-07

**Authors:** Giovanna Garufi, Luisa Carbognin, Armando Orlandi, Antonella Palazzo, Giampaolo Tortora, Emilio Bria

**Affiliations:** ^1^ Oncologia Medica, Fondazione Policlinico Universitario Agostino Gemelli Istituto di Ricovero e Cura a Carattere Scientifico (IRCCS), Roma, Italy; ^2^ Università Cattolica Del Sacro Cuore, Roma, Italy; ^3^ Division of Gynecologic Oncology, Department of Woman and Child Health and Public Health, Fondazione Policlinico Universitario Agostino Gemelli IRCCS, Roma, Italy

**Keywords:** breast cancer, bone marrow metastasis, visceral crisis, endocrine therapy, CDK4/6 inhibitor, case report

## Abstract

The efficacy and safety of the combination of endocrine therapy (ET) and CDK4/6 inhibitors for patients with hormone receptor (HR)-positive HER2-negative metastatic breast cancer (BC) presenting with visceral crisis or life-threatening conditions represent a challenge for daily clinical practice. Indeed, the peculiarity of this clinical presentation (signs and symptoms of rapidly progressive disease) does not allow to include such patients in a trial aiming for drug approval. On the basis of the scientific evidence available so far, chemotherapy represents the standard of care according to guidelines, on the basis of the more rapid activity in comparison with ET alone. Besides, the combination of ET and CDK4/6 inhibitors have demonstrated in clinical trials to have clinically impactful activity in a short time, thus suggesting a potential role in advanced tumors that require rapid response. Herein, we report the clinical history of a young woman with HR-positive HER2-negative metastatic BC and a pancytopenia due to carcinomatosis of the bone marrow receiving letrozole and leuprorelin plus the CDK4/6 inhibitor palbociclib, who significantly derived clinical benefit from treatment. Considering that these peculiar cases are excluded from clinical trials, the estimation of the magnitude of the benefit of the newer ET combination may potentially represent a practical question for large case series and real-world studies.

## Introduction

Breast cancer (BC) is the most common cancer in women (1) and the lifetime probability of developing invasive BC in women from birth to death is 12.8% (2). Systemic treatment for early and advanced BC is based on the expression of estrogen receptors (ERs), progesterone receptors (PRs), and human epidermal growth factor receptor 2 (HER2) (3). The most common subtype of BC is represented by the luminal, that is, hormone receptor (HR)-positive, HER2-negative disease, accounting for about 70% of all cases (4). In early and metastatic setting, improvements in survival outcomes have been achieved, following the development of new drugs (1).

Regarding the metastatic setting, the standard of care in the first-line therapy in advanced luminal breast cancer (LBC) includes endocrine treatment (ET) alone or in combination with a targeted therapy for patients without visceral crisis, defined as severe organ dysfunction, as assessed by signs and symptoms, laboratory studies, and rapid progression of disease (5). The choice of the first-line ET depends on the disease’s presentation (*de novo* metastatic or recurrence after neoadjuvant/adjuvant treatment), on the type of neoadjuvant/adjuvant ET, if any, and whether relapse occurs while under adjuvant ET or after the end of adjuvant ET. Patients are defined as endocrine sensitive if they have never been exposed to ET or relapsed more than 1 year after completion of adjuvant ET (5). For patients with *de novo* metastatic disease or presenting more than 12 months after completion of adjuvant aromatase inhibitor (AI), first-line ET standard of care includes AI plus a cyclin-dependent kinase 4 and 6 (CDK4/6) inhibitor or fulvestrant plus a CDK4/6 inhibitor, while tamoxifen, AI alone, or fulvestrant alone are reserved for selected cases (5). For patients with disease recurrence during adjuvant ET or less than 12 months after completion of adjuvant ET, ET options include fulvestrant plus a CDK4/6 inhibitor or fulvestrant alone, AI, or tamoxifen in selected cases (5). With AI or fulvestrant alone as first-line treatment, it is possible to obtain a median progression-free survival (PFS) of just over a year (6).

CDK4/6 inhibitors are the most recently introduced class of agents for the treatment of advanced LBC (7). The dysregulation of the cyclin D-CDK4/6 pathway has been implicated in BC biology (8–10). The CDK4/6 enzymes are serine/threonine kinases, the activity of which is modulated by the interaction with a cyclin regulatory subunit (11). They are involved in cell cycle progression, playing a key role in cell proliferation (12). Cyclins of the D class (D1, D2, and D3) are regulators of the CDK4 and CDK6, with which they form active complexes (13). Cyclin D1, which is overexpressed in approximately half of BCs, is a transcriptional target of the ER (9, 14). The ER signaling increases cellular levels of the D class cyclins, particularly cyclin D1, inactivating the retinoblastoma (Rb) tumor suppressor protein and leading to cell cycle progression, overcoming the G1/S transition phase (15).

Endocrine therapy inhibits activation of the cyclin D-CDK4/6 signaling pathway, while CDK4/6 inhibitors induce cell cycle arrest in Rb-proficient cells (16). Palbociclib, ribociclib, and abemaciclib have been approved in combination with AIs (letrozole or anastrozole) or fulvestrant. In the endocrine-sensitive setting, they showed an improvement of PFS of approximatively 10 months, from 14–16 months to ≥24 months, compared to hormone monotherapy with AIs, as demonstrated in their pivotal trials PALOMA-2, MONALEESA-2, and MONARCH-3 (17–19). In the same setting, MONALEESA-7 trial showed a similar clinical efficacy of ribociclib in combination with ovarian suppression and tamoxifen or AI in premenopausal women (20). However, regulatory agencies have not approved the combination of tamoxifen and ribociclib, because of QT prolongation risk (21). While for PALOMA-2, MONALEESA-2, and MONARCH-3 trials, overall survival (OS) data are not yet reported, in the MONALEESA-7 study, the addition of ribociclib also significantly improved OS compared with placebo: at 42 months follow-up, 70.2% in the ribociclib group and 46.0% in the placebo group were alive (hazard ratio [HR] 0.71; *p* = 0.00973) (20). In the endocrine-resistant setting, the addition of a CDK4/6 inhibitor approximately doubled PFS compared to fulvestrant alone, as demonstrated in PALOMA-3, MONALEESA-3, and MONARCH-2 trials (22–24). While the PALOMA-3 trial reported a numerical increase in OS in favor of the experimental arm from 28 to 34.9 months without a statistical significance (HR 0.81; *p* = 0.09) (22), in the MONARCH-2 and MONALEESA-3 studies, the addition of the CDK4/6 inhibitor to ET resulted in OS improvement as well, in addition to PFS (23, 25). However, all these pivotal studies did not include patients with visceral crisis or with signs and symptoms of rapidly progressive disease.

In patients with advanced LBC, chemotherapy is indicated for the treatment of visceral crisis or significant functional organ impairment and when it is no longer possible to consider the endocrine-responsive disease (5, 26). Important organ compromise leads to a clinical indication for more rapidly effective therapy, especially since another treatment option at progression is likely not possible (5). Disseminated carcinomatosis of the bone marrow (DCBM) is a life-threatening condition, potentially leading to a visceral crisis, for which systemic chemotherapy is recommended. Here, we present a case of a LBC patient with a DCBM at the diagnosis, treated with a combination of ET and CDK4/6 inhibitor as first line therapy.

## Case Description

**Figure 1:**
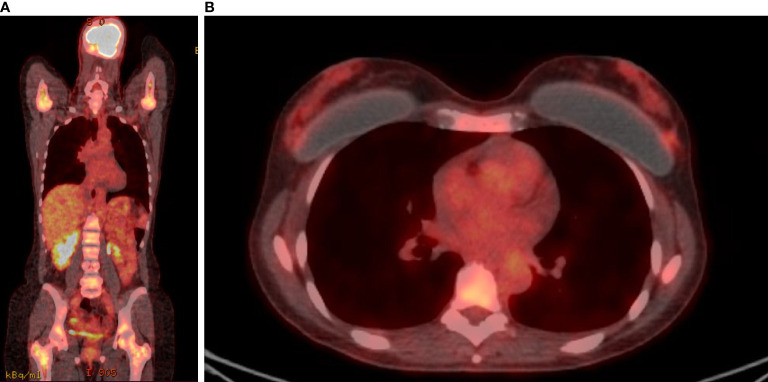
FDG-PET/CT. FDG uptake was detected in almost all bone segments **(A)** and in breast lesions **(B)** before treatment.

A 46-year-old premenopausal woman had a past medical history of 1-cm papillary thyroid cancer (pT1a) diagnosed at the age of 40 and treated with total thyroidectomy, uterine fibromatosis treated with hysterectomy and bilateral breast augmentation. She was diagnosed in August 2018 with metastatic bilateral infiltrating lobular carcinoma of the breast. In June 2018, she presented with general fatigue. Blood tests performed in August 2018 revealed severe anemia (hemoglobin level, 7.5 g/dl), which required red blood cell transfusions, and a reduction in white blood cell and platelet counts (white blood cell count 2,900/μl; platelet count, 110,000/μl). Other laboratory studies showed elevated alkaline phosphatase. A bone marrow biopsy was performed; pathology revealed that poorly differentiated lobular carcinoma with strong immunohistochemical (IHC) expression of ER (80%) had infiltrated the bone marrow, with reduced numbers of the three hemopoietic cell lines (myeloid, erythroid, and megakaryocytic). An 18-fluorodeoxyglucose-positron emission tomography computed tomography (FDG-PET/CT) showed uptake with activation of the bone marrow in all bone segments, a nuanced uptake in bilateral breast and a pelvic ascitic effusion (**Figures 1A**, **B**). Mammography, ultrasonography, and magnetic resonance imaging of the breast found a bilateral multicentric and multifocal disease, consistent with the lobular histotype, with bilateral axillary enlarged lymph nodes. She underwent fine needle biopsy of a suspicious nodule in the right breast, which revealed a moderately differentiated invasive lobular carcinoma. The IHC test proved positive for ER (80%) and PR (80%) and negative for HER2 (1+). The Ki67 labeling index was low (10%). Serum levels of CA15.3 were elevated (46 U/ml).

The patient had been suggested to start chemotherapy. However, she was concerned about alopecia and chemotherapy-related side effects in general. Thus, the patient had approached our center for a second opinion. We kept in mind the risks that chemotherapy carried due to the low blood cell count. On the other hand, we were not confident enough to start endocrine monotherapy. We considered the encouraging survival results, together with the not relevant hematologic toxicity, of the combination of ET with the CDK4/6 inhibitor palbociclib. We shared with the patient the possibility of undertaking enhanced ET, adding a new drug recently available in clinical practice, palbociclib, specifying that no data were available on the efficacy and safety of the proposed combination in similar clinical situations. Therefore, we agreed with the patient to start ET with a combination of an AI, letrozole, and a CDK4/6 inhibitor, palbociclib, switching to chemotherapy in case of worsened signs and symptoms of the metastatic disease. We also added ovarian suppression with a luteinizing hormone-releasing hormone agonist (LH-RHa) as the patient was premenopausal.

In September 2018, she started palbociclib 125 mg orally daily 3 weeks on and 1 week off, letrozole 2.5 mg orally daily continuously and leuprorelin 3.75 mg intramuscular injections every 28 days. The patient tolerated treatment well, except for grade 1 fatigue, arthralgia, and hot flashes. After the first two weeks of therapy with palbociclib, the patient also had grade 4 neutropenia without fever, which resolved with discontinuation of palbociclib for 3 weeks. Then, the CDK4/6 inhibitor was restarted at the reduced dose of 100 mg daily, with subsequent persistent asymptomatic grade 2 neutropenia.

**Figure 2:**
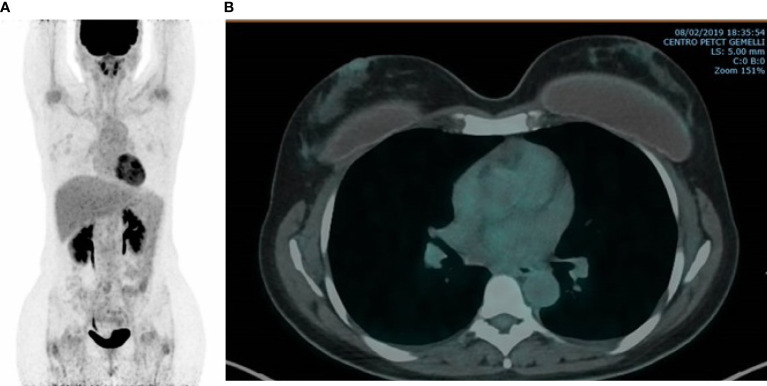
FDG-PET/CT. Completely disappeared bone **(A)** and breast **(B)** uptake was observed after five months of AI, LH-RHa and CDK4/6 inhibitor.

**Figure 3:**
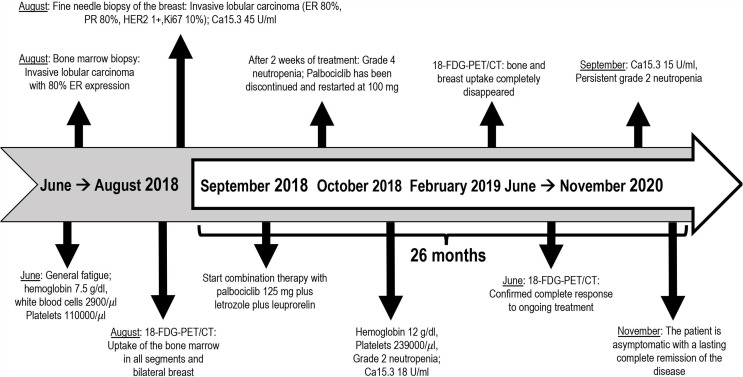
Relevant clinical data and therapies from diagnosis till now organized as a timeline.

After 4 months of treatment, her red blood cells and platelet count was restored to within the normal range (hemoglobin level 12 g/dl, platelet count 239,000/μl) with grade 2 neutropenia being treated with palbociclib. Serum levels of Ca15.3 decreased to 18 U/ml. In February 2019, follow-up PET/CT scan showed the resolution of the hypermetabolic breast tumors and bone metastatic foci with the disappearance of the ascitic effusion (**Figures 2A, B**). Patient’s subsequent PET/CT imaging every 6 months confirmed complete response to ongoing treatment with last PET/CT performed in June 2020. Last tumor markers over the past 20 months were negative until September 2020. So far, the patient is clinically asymptomatic for metastatic BC and she remains in a durable complete remission for 26 months on this treatment regimen. Timeline of relevant clinical data and therapies are presented in **Figure 3**.

## Discussion

In the current clinical case, the patient presented at the diagnosis with a lobular carcinoma of the breast and a diffuse infiltration of the bone marrow by malignant cells. Bone marrow metastasis from solid tumors is frequent in breast and prostate cancer and gastric adenocarcinoma and can lead to hematological disorders such as thrombocytopenia, anemia, and leukopenia. In particular, anemia and thrombocytopenia are usually the first clinical symptoms in patients with DCBM (27). Although micrometastatic spread of BC cells to the bone marrow has been described in up to one-third of patients with early stage at the diagnosis and it is a negative prognostic factor for recurrence (28), the development of clinically relevant marrow involvement represents a rare manifestation (27).

Few data are available on the epidemiology, diagnosis, treatment, and outcome of BC patients affected with diffuse bone marrow metastasis. BC patients with DCBM usually presented at the diagnosis with moderately or poor differentiated, multicentric, HR-positive tumors and invasive lobular histology, like the patient described in our clinical case. In particular, the multicentric primary disease is a strong predictor for development of earlier DCBM (29). PET/CT imaging has been proposed as a diagnostic tool, but the pathological examination of a bone marrow biopsy and/or aspirate is considered the gold standard for the diagnosis of DCBM and for the treatment decision (30). Indeed, IHC features in terms of HRs and HER2 of the primary BC tumors and metastatic tissues may differ because of the genetic heterogeneity of the cancer cells. Therefore, the histopathological confirmation of the metastatic lesion IHC characteristics should be performed whenever possible (31).

The treatment of DCBM is challenging because of cytopenia and the prognosis is poor: the median OS being approximately 19 months from the diagnosis of DCBM, regardless of the interval between the diagnosis of DCBM and the first diagnosis of BC. Although DCBM is typically characterized by cytopenia, systemic treatment can be administered with the purpose of a long-lasting disease control, despite an increased risk of hematological complications (27), without differences in disease control and median survival between single agent chemotherapy and polychemotherapy regimens (29).

According to international guidelines, the initial treatment of HR-positive, HER2-negative advanced BC involves the administration of ET, even in the presence of visceral disease, excluding those patients with life-threatening conditions or with visceral crisis as presentations of the disease (5, 26). Palbociclib, ribociclib, and abemaciclib are third-generation CDK4/6 inhibitors and represent an established therapeutic class for metastatic LBC. Preliminary results show that a combination of ET with a CDK4/6 inhibitor provides a superior clinical benefit in terms of PFS compared to chemotherapy in patients with premenopausal metastatic LBC. The Korean phase II Young-PEARL trial randomized 184 premenopausal patients to a combination of exemestane, LH-RHa, and palbociclib or capecitabine; approximately half of the patients were treatment naïve in the advanced setting. The study showed that ET combination led to a significantly longer median PFS compared with chemotherapy arm in premenopausal patients pretreated with tamoxifen (20.1. *vs*. 14.4 months; HR 0.659; *p* = 0.0235) (32). A similar PFS was found in the phase III PEARL trial, which tested the combination of ET and palbociclib *vs*. capecitabine in postmenopausal patients pretreated with aromatase inhibitors (median PFS was 7.5 *vs*. 10 months for fulvestrant plus palbociclib *vs*. capecitabine; HR 1.09; *p* = 0.537) (33). Ongoing studies are also exploring the comparison of ET with a CDK4/6 inhibitor and chemotherapy in endocrine-sensitive LBC patients. A randomized, open-label, phase IV study evaluating ET plus palbociclib *vs*. chemotherapy in metastatic LBC patients in a real-world setting (PADMA study, NCT03355157) is currently being conducted. The objective of the trial is to demonstrate an improvement in time to treatment failure with endocrine combination therapy compared to chemotherapy, in order to provide real-world evidence that palbociclib plus ET is the better first-line choice not only compared to ET, but also compared to chemotherapy. Noteworthy, the combination of ET plus a CDK4/6 inhibitor is also tested in comparison with chemotherapy in the setting of significant visceral impairment. The RIGHT Choice study (NCT03839823) is a phase II randomized trial that is exploring ET plus ribociclib compared with chemotherapy in terms of PFS in non-pretreated premenopausal LBC patients with symptomatic visceral metastases, or rapid progression of disease or impending visceral compromise.

Studies regarding the efficacy and safety of ET, with or without CDK4/6 inhibitors, in LBC with DCBM are extremely limited because patients with visceral crisis or life-threatening conditions, such as bone marrow infiltration, are excluded from clinical randomized trials. In relation to our clinical case, chemotherapy, due to its rapid time to response, represents an important therapeutic resource, even as a less toxic and better tolerated weekly administration, and it is generally recommended for the treatment of BC with DCBM (27, 29, 30). In general, in the presence of visceral crisis or proof of ET resistance for advanced LBC, both combined and sequential single-agent chemotherapy are reasonable options. Nevertheless, given the overlapping survival results, sequential monotherapy is recommended as the preferred choice, reserving combination chemotherapy for patients with rapid clinical progression, life-threatening visceral metastases, the need for rapid symptom, and/or disease control or visceral crisis.

However, in the current case, due to its cytotoxic effect, chemotherapy may initially worsen hematological disorders due to pre-existing marrow infiltration by cancer cells. The hematological chemotherapy toxicity sometimes requires supportive care, such as transfusion of red blood cells and platelets and the administration of granulocyte growth factors. In contrast, ET, although less rapid than chemotherapy in obtaining a disease response, is better tolerated and has no hematological toxicity. Therefore, endocrine agents, from the point of view of toxicity, can be used safely in the treatment of BC with DCBM (34).

There are few published reports in the literature on the efficacy of ET in the treatment of LBC with DCBM. The case of a 64-year-old woman who presented at the diagnosis with diffuse bone marrow metastasis from an occult HR-positive, HER2-negative BC was described; tamoxifen treatment achieved a sustained response, with normalization of hematological parameters (35). At our knowledge, only one clinical case has been published on the use of ET with CDK4/6 inhibitors in a 59-year-old patient with a DCBM from an occult LBC. The patient was treated initially with letrozole and then with a combination of fulvestrant and palbociclib as second-line treatment, resulting in a response to treatment lasting more than 1 year. Palbociclib was initiated at a reduced dose (75 mg/day), being concerned about a possible exacerbation of pre-existing hematological toxicity (34).

Our premenopausal patient was treated with an ET regimen containing letrozole, leuprorelin, and palbociclib standard dose, achieving a complete metabolic response that has lasted for more than 2 years. We chose to start with the highest dose level of palbociclib (125 mg/day) because we were aware that the hematological toxicity of CDK4/6 inhibitors is due to their cytostatic but non-cytotoxic effect on the blood marrow cells and therefore the hematological adverse events are generally low grade; while neutropenia is frequent, febrile neutropenia occurs only in 1%–1.8% among patients treated with CDK4/6 inhibitors *vs*. 0%–1% of patients treated with ET alone (17–19, 36–39), and it does not need granulocyte colony-stimulating factors. Nevertheless, grade 4 neutropenia occurred during the first month of therapy, and we temporarily interrupted palbociclib for 3 weeks, restarting treatment at the first level of dose reduction (100 mg/day), without complications. She currently continues to receive safely this ET combination.

At present, there are three commercially available CDK4/6 inhibitors for the metastatic LBC treatment: palbociclib, ribociclib, and abemaciclib (21). Palbociclib is the first in class approved CDK4/6 inhibitor. In the endocrine-sensitive setting, the three CDK4/6 inhibitors were tested in the pivotal studies PALOMA-2, MONARCH-3, MONALEESA-2, and MONALEESA-7 (17–19, 38). While in the PALOMA-2, MONALEESA-2, and MONARCH-3 trials, only postmenopausal patients were enrolled (17–19), the MONALEESA-7 study included only premenopausal patients, showing that the addition of ribociclib to a combination of tamoxifen or AI and ovarian suppression results in median PFS improvement of about 10 months (from 13 to 23.8 months; hazard ratio 0.55; *p* < 0.001) (38), then confirmed by the efficacy in median OS (20). Furthermore, abemaciclib was the last of the CDK4/6 inhibitors to be approved by the FDA and EMA. The MONARCH-2 and MONARCH-3 pivotal studies showed that abemaciclib, due to its different pharmacodynamic profile, has less hematological adverse events than palbociclib and ribociclib, with grade 3/4 neutropenia rates approximately 20% compared to 60% for palbociclib and ribociclib (21). Given the premenopausal results of ribociclib and the low incidence of hematological toxicity of abemaciclib, these two drugs could be preferred for the treatment of this patient. However, at the time of diagnosis, the only drug we could have access to was palbociclib.

In conclusion, the combination of ET and CDK4/6 inhibitor may provide longer clinical benefit than chemotherapy in advanced LBC, with better response rates and time to response similar to chemotherapy. On the other hand, the combination of ET and a CDK4/6 inhibitor is less toxic and leads to better quality-of-life outcomes than chemotherapy. However, the population treated in daily clinical practice often differs from that included in randomized controlled trials, such as the patient in our clinical case. Indeed, there are no prospective data on the use of CDK4/6 inhibitors in patients with visceral crisis or rapidly developing life-threatening clinical manifestations. For this reason, it is difficult to conclude on any specific treatment recommendation in these patients. Therefore, the publication of case reports, case series, or real-word studies should be encouraged, because they help to clarify the efficacy and safety of innovative drugs, saving or delaying the use of chemotherapy, even in borderline clinical situations.

## Data Availability Statement

The original contributions presented in the study are included in the article/supplementary material. Further inquiries can be directed to the corresponding author.

## Ethics Statement

Written informed consent was obtained from the relevant individual(s), and/or minor(s)’ legal guardian/next of kin, for the publication of any potentially identifiable images or data included in this article.

## Author Contributions

GG and EB conceived the idea of the case report. GG drafted the manuscript, and LC and EB critically reviewed it. GG, AO, AP, and EB were involved in the patient’s clinical management. All authors contributed to the article and approved the submitted version.

## Conflict of Interest

The authors declare that the research was conducted in the absence of any commercial or financial relationships that could be construed as a potential conflict of interest.

## Publisher’s Note

All claims expressed in this article are solely those of the authors and do not necessarily represent those of their affiliated organizations, or those of the publisher, the editors and the reviewers. Any product that may be evaluated in this article, or claim that may be made by its manufacturer, is not guaranteed or endorsed by the publisher.
